# Development and Characterization of a Probe Device toward Intracranial Spectroscopy of Traumatic Brain Injury

**DOI:** 10.1021/acsbiomaterials.0c01156

**Published:** 2021-02-22

**Authors:** Max Mowbray, Carl Banbury, Jonathan J. S. Rickard, David J. Davies, Pola Goldberg Oppenheimer

**Affiliations:** † Department of Chemical Engineering and Analytical Science, University of Manchester, The Mill, Sackwville Street, Manchester M1 3AL, U.K.; ‡ School of Biochemical Engineering, EPS, University of Birmingham, Edgbaston, Birmingham B15 2TT, U.K.; § Department of Neuroscience and Ophthalmology, Institute of Inflammation and Ageing, National Institute for Health Research, Queen Elizabeth Hospital Birmingham, University of Birmingham, Mindelsohn Way, Birmingham B15 2TH, U.K.; ∥ Department of Physics, Cavendish Laboratory, University of Cambridge, JJ Thomson Avenue, Cambridge CB3 0HE, U.K.; ⊥ Healthcare Technologies Institute, Institute of Translational Medicine, Mindelsohn Way, Birmingham B15 2TH, U.K.

**Keywords:** Raman device, intracranial spectroscopy, SKiNET, traumatic brain injury biochemistry

## Abstract

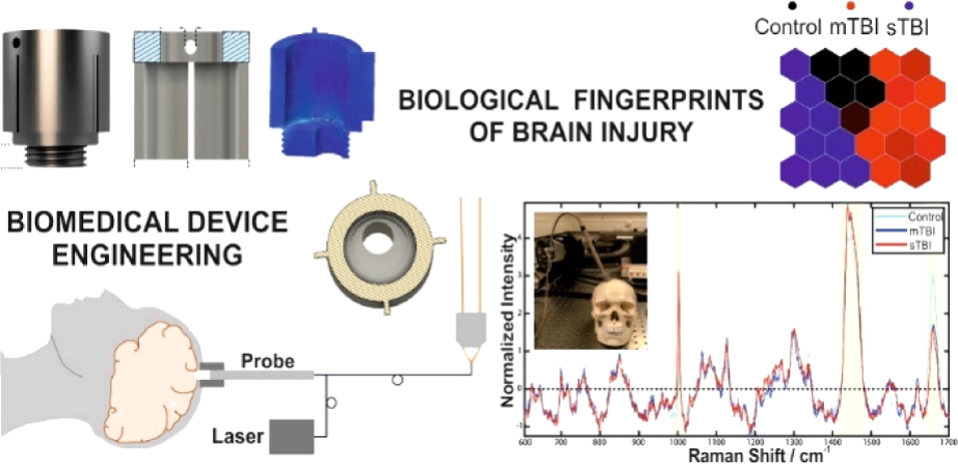

Traumatic brain injury is a leading cause of mortality worldwide, often affecting individuals at their most economically active yet no primary disease-modifying interventions exist for their treatment. Real-time direct spectroscopic examination of the brain tissue within the context of traumatic brain injury has the potential to improve the understanding of injury heterogeneity and subtypes, better target management strategies and organ penetrance of pharmacological agents, identify novel targets for intervention, and allow a clearer understanding of fundamental biochemistry evolution. Here, a novel device is designed and engineered, delivering Raman spectroscopy-based measurements from the brain through clinically established cranial access techniques. Device prototyping is undertaken within the constraints imposed by the acquisition and site dimensions (standard intracranial access holes, probe’s dimensions), and an artificial skull anatomical model with cortical impact is developed. The device shows a good agreement with the data acquired *via* a standard commercial Raman, and the spectra measured are comparable in terms of quality and detectable bands to the established traumatic brain injury model. The developed proof-of-concept device demonstrates the feasibility for real-time optical brain spectroscopic interface while removing the noise of extracranial tissue and with further optimization and *in vivo* validation, such technology will be directly translatable for integration into currently available standards of neurological care.

## Introduction

Rapid *in vivo* healthcare point-of-care diagnostics are of critical importance to clinical medicine. They aid in providing optimized, individually tailored, efficient treatment. Currently, no primary disease modifying interventions exist for the treatment of traumatic brain injury (TBI).^1,2^ Clinical management is primarily directed toward the normalization of the intracranial environment, be it pressure, perfusion, or oxygenation.^2^ The maintenance of effective intracranial homeostasis is aimed at minimizing secondary brain injury and providing the most favorable conditions for the preservation of neurological tissue. Invasive (placed into brain tissue) monitoring modalities including pressure transducers and oxygen tension sensors are used to provide data to support the maintenance of such favorable physiological conditions. As independent parameters, both intracranial pressure (ICP) and brain tissue oxygen-tension guided intervention provide minimal improvement in outcome after severe TBI.^3,4^ The pathophysiology of injury evolution is multi-faceted and currently not understood well enough to provide effective pharmacological targeting to reduce injury burden or progression. Surgical intervention has demonstrated a role in saving lives; however, it is unclear as to what extent the outcome is improved in individuals who survive.^5,6^ One of the reasons often attributed to the modest improvements in outcome observed after surgical intervention is the course stratification systems currently in place for classifying TBI. The Glasgow Coma Scale (GCS) and Marshall CT grading system are among the most common grading tools used to quantify the severity of injury burden.^7^ Through these tools (along chiefly with ICP measurement), interventions and management plans are formulated. It is often postulated that a more in-depth understanding of the biochemical injury evolution within individual patients may allow existing interventions to be used more effectively, along with the identification of novel pharmacological and non-pharmacological therapeutic targets. Quantitative measurement of a patient’s disease burden *via* condition specific biomarkers is crucial in modern practice to guide tactful therapeutic treatment.^8^ Disease-indicative biomarkers provide insights into the biological pathways underpinning certain pathologies allowing effective stratification and classification of pathology for the purpose of guiding management.^9^ However, typical time-to-results of many diagnostic and prognostic tests specific to TBI such as diffusion tension magnetic resonance imaging (MRI) and neuro-cognitive assessment (NAB battery) can be greater than 13 weeks,^10^ with as much as 10 weeks being a common representative time.^11^ In the scope of critical care, such untimeliness provides a barrier to effective management guidance.

TBI constitutes a major burden to healthcare services. Globally, it is the most common cause of death in those below 45 years of age. In 2017, there were 531 admissions with a TBI per 100,000 individuals in the UK alone.^1,12^ These numbers however are typically only inclusive of the cases that present to secondary care. If cases that did not present to emergency departments are included, the actual incidence of TBI in developed nations is around 790 cases per 100,000 per year.^13^ The emotional and functional impacts on patients and their families are life changing, with large subsequent direct and indirect costs associated with acute and community care.^14^ However, the real cost of injury is far further in reach, with the life medical, educational, and social cost of care for a pediatric case of TBI estimated to be in the region of £4.9 million,^15^ with the total annual cost globally in the region of £43 billion.^16^ The pathophysiology of TBI is complex and broadly separated into primary and secondary insults. The primary insult is underpinned by a momentum transfer to the brain from either the direct cause of injury, for example, a strike to the head or the skull itself.^17^ The secondary insult is considerably more complex. It is a pathophysiological cascade similar to a chain reaction. Many of the pathologies detailed are interrelated, with one proliferating the other. For example, a decoupling of cerebral blood flow and metabolism propagates oxidative stress and excessive glutamatergic excitation, which can lead to neuro-inflammation and programmes of cell death. In the context of severe TBI, these processes often lead to cerebral swelling and raised ICP. Cerebral oedema (CO) evolving as a result of secondary insult plays a key role in the development of raised ICP. Other pathological processes may occur concurrently, such as hydrocephalus, where the accumulation of cerebrospinal fluid (CSF) contributes to high ICP, along with the distortion of important neuro anatomical structures leading to tissue injury.^18^ Although it is well-established that the ICP is a key target for intervention and management, current options often confront the specific parameter of abnormal ICP and neglect the underlying biological pathways, such as in CO. In such cases, a craniotomy or CSF diversion is used to reduce pressure—providing volume for the brain to swell and limiting any increase in the ICP, although not addressing the underlying causes that have led to this situation.

In the past decade, several serum biomarkers have been showing promise as potential candidates to provide insights into the pathophysiology of TBIs and their associated neurodegeneration, particularly S100B, UCHL1, NAA, and NFP.^19−22^ Some biomarkers that have demonstrated robust correlation with TBI outcomes have been identified in difficult-to-sample fluids such as CSF. Within this fluid, numerous biomarkers have demonstrated exciting utility, specifically correlating with severity of injury as evaluated by the GCS.^23^ The *in vitro* analysis of serum and CSF samples of 98 serum biomarkers in one investigation correlated with the level of severity, with 49 of which predicting the patients’ outcome.^23^ Studies such as this demonstrate the potential of use of biofluid-based assays and sensing methods. The clear advantages of point-of-care diagnostics for acute diseases and especially TBI ultimately offer guidance for tactful healthcare management. Specifically, within the context of TBI, a clearer understanding of when to utilize current pharmacological and surgical intervention would be of great value. Multiple previously trailed interventions, which demonstrated promising pre-clinical effect failed to translate this into tangible improvements in outcome in controlled trials.^23^ More specific pathology-based stratification or classification of injuries may allow better sub-group targeting of these therapeutics and produce the improvements in clinical outcome that was anticipated.

Concurrently, Raman spectroscopy is a sensitive analytical method, which provides a unique spectral fingerprint of target analytes *via* the inelastic scattering of light. In contrast to the *in vitro* bioassays, its portability enables reliable *in vivo* point-of-care diagnostics to be conducted. Raman spectroscopy can offer a label-free mechanism for measuring changes to biochemistry, which can be applied *in vivo* in invasive settings such as surgery, which has also shown promise for non-invasive measurements. In neurology, there has been an emphasis on using Raman spectroscopy for cancer detection, as an interoperative guide during tumor resection.^24−26^ This was motivated to a degree by the diffuse nature of glioblastomas, where discrepancies between preoperative MRI scans lead to relapse from the resection margin. Further, the availability of commercial portable Raman devices for industrial applications enables the proof-of-concept interventions using Raman spectroscopy as well as the relatively straightforward integration into existing surgical practice and healthcare pathways. This concept is further highlighted across several publications by Jermyn *et al.*,^25,27,28^ where the probe developed featured a compact concentric design, with a central excitation illumination fiber and peripheral collection fibers, which allowed for its integration into the existing surgical practice. By comparison to MRI, the use of Raman spectroscopy showed a greater specificity and sensitivity, centimeters beyond the region of detection using the radiological scans. Following the initial study, the researchers performed *ex vivo* validation that combined several different animal models and devices. Desroches *et al.*(24) extended the initial concept to show that high wavenumber bands alone can be used in the classification of cancerous tissue. The authors also have shown that the need to manually subtract background effects caused by fluorescent lights in the operating theatre can be ignored by using machine learning.^28^ Raman spectroscopy was also applied to study the different brain structures, with clear differences observable in the spectra of white matter and gray matter. The work by Kast *et al.*(29) showed that Raman bands displaying key changes for white matter and gray matter come from a mixture of lipids and proteins. A similar analysis was performed by Koljenović *et al.*,^30^ using falsely colored Raman image maps of tissue from both brain and bladder in a porcine model. The authors noted design considerations when using fiber optics, which allowed for the bulk laser and the spectrometer setup to be kept away from tissue and patient measurements as discussed previously in the work by Desroches *et al.*(24) Raman spectroscopy has been shown to be further sensitive to conformational changes in proteins, which have important implications for neurodegenerative diseases such as Alzheimer’s and Parkinson’s. Ji *et al.*(31) showed a blueshift of 10 cm^–1^ to the amide I band as a result of misfolded proteins in a murine model of Alzheimer’s disease. Flynn *et al.*(32) studied the formation of αsynuclein amyloid fibrils associated with Parkinson’s disease, as they aggregate from solution under differing pH levels and for mutant forms of the protein linked to early onset of the disease. The first experiment showing that Raman spectroscopy is sensitive to biochemical changes following TBI was performed by Tay *et al.*(33) using a focal brain injury model (left motor cortex) in mice. Dramatic changes were observed in the spectral profile in response to injury, using the uninjured hemisphere as a control. Although the Raman analysis in this study was purely qualitative, immunohistology studies were also performed, showing that the observed changes may be linked to mitochondrial activity and apoptosis following injury.^33^ More recent work by Surmacki *et al.*(34) has studied the temporal response to TBI at 2 and 7 days after injury in a murine model. In addition to changes from the hemisphere of injury, spectral changes were also observed from the contralateral hemisphere and seen to evolve with time. Unlike the previous study, attribution to the underlying biochemistry was made with reference to Raman spectra of brain specific lipids to make qualitative attribution to a relative increase in cholesterol with injury. Recently, Raman spectroscopy combined with advanced machine learning was applied to investigate whether the retina can reflect the brain microenvironment after TBI,^39^ in a clinically relevant murine model, showing that spectra from the eye can distinguish moderate TBI and severe TBI from a sham group, and show this to be as a result of similar chemical changes to those seen at the point of injury on the brain with the detected changes identified being largely due to metabolic distress and the release of cardiolipin, consistent with the use of mass spectrometry as a diagnostic modality for TBI.^35^ While mass spectrometry can provide superior molecular discrimination, Raman spectroscopy has the advantage of being both a portable and nondestructive technique with many ongoing efforts in the field for translation into *in vivo* measurements and diagnostics.

Raman spectroscopy, therefore, enables rapid and specific quantitative analysis of chemical composition and structure, requires no complex sample preparation or defined sample size with an inherent ease of detection in aqueous conditions.^36,37^ Of an importance, is its non-destructive nature, posing it as ideal for use in clinical settings where deploying Raman-based technology can enable differentiation between disease and healthy tissue states during the neurosurgery.^38^ As such, in conjunction with the emergence of state-of-the-art machine learning techniques, the development of reliable, rapid Raman-based *in vivo* diagnostic tools ultimately promises better quality of patient treatment. While Raman spectra define a biochemical fingerprint, uniquely determined by the underlying molecular constituents, the spectral interpretation due to the biological samples’ complexity is not trivial. Recently, we developed a machine learning technique based on the self-optimizing Kohonen index network (SKiNET) for simultaneously providing rich information and classification from complex biological matrices, even with noisy or poor-quality spectra with very subtle differences that would result from a low laser power and short acquisition times required in intraoperative or diagnostic applications of Raman spectroscopy.^39^

Herein, we report the development of a Raman probe-based device, which utilizes the specificity of inelastic scatter in conjunction with access to CSF and brain tissue provided by standard cranial access techniques^40^ undertaken in approximately 15–20% of TBI admissions to secondary care.^41^ The purpose of which would be to temporally track the pathology and injury evolution of moderate to severe TBI (for which intracranial access is mandated), generating a “real-time” brain optical interface. Using three-dimensionally printed intracranial bolt with mechanical stress test simulations for auto tapping thread, a prototype device is developed for these purposes and tested using anatomically correct skull model. The design incorporates off the shelf Raman probe, and spectral response is demonstrated *via* the direct comparison to commercial Raman system. Single-use disposable bolts with quartz windows permit reusable procedure of the probe. This Raman device combined with our versatile SKiNET algorithm enables the capability to discern between TBI and healthy groups. The self-organizing map (SOM) discriminant index (SOMDI) for each injury state uniquely provides further insights into the biological spectral features responsible for clusters observed, and the regression fitting adds further depth allowing to quantitatively attribute changes in the underlying biochemistry. The developed proof-of-concept device demonstrates the feasibility for real-time optical brain spectroscopic interface removing the noise of extracranial tissue, which after successful optimization, *in vivo* validation, and clinical trial can be translatable into current standard of care. The results from this study thus lay a platform to aid in the long-term the tactful therapeutic management, which can also provide important clinical insights into the pathophysiological cascades. This in turn will further enable better-quality post neurotraumatic care as well as the development of novel pharmaceutical and physical therapies.

## Materials and Methods

### Materials

Fabrication of device prototypes was undertaken using RS PRO 2.85 mm natural poly(vinyl alcohol) (PVA) 3D printer filament, RS PRO 2.85 mm black acrylonitrile butadiene styrene (ABS) 3D printer filament, and RS PRO 2.85 mm white ABS 3D printer filament. The PVA filament was used exclusively in the generation of support during the printing process. Polystyrene was purchased from Sigma-Aldrich. The polished disc was acquired from Robson Scientific. Murine species subjected to craniotomy following induction of controlled cortical impact injury are described in refs.^39,42^ Three days after the TBI the brain was removed and post-fixed in sodium chloride saline and the cortex (neocortex) tissues were used for spectroscopic studies. The ethical framework for the laboratory (*in vitro*) consists of the Home Office license for the sacrifice of murine species (Sprague Dawley rats). The clinical and iCal frameworks comprise the red diamond ethics (ERN_17-0916) part of the NIHR portfolio study.

### Design and Fabrication of 3D Printed Elements

The fabrication of the 3D printed elements detailed utilized Autodesk Fusion 360 (AF360) software. AF360 is an integrated computer-aided design (CAD), engineering (CAE), and manufacturing software, which in conjunction with Cura software and an Ultimaker S3 fused filament fabrication (FFF) printer facilitates rapid design, preliminary testing, and manufacture of prototype components. To ensure against the risk of infection, the device prototype was designed to seal the cerebral matter from the external environment. This was facilitated by inclusion of a 10 mm diameter quartz polished disc.

### Finite Element Analysis

Finite element analysis (FEA) was conducted through the CAD and CAE tools featured within AF360. Such tools are broadly encompassed by the fields of FEA and computational fluid dynamics. The AF360 software enabled the inherent ease of FEA under its simulation feature. Implementation of the tests followed specific configurations, underpinned by constraints, loads, and contacts (friction) as following: all comparative simulations proceeded on the basis of the same constraints and load; and, in AF360, implementation of constraints was carried out with the aim of ensuring the static stability of the model, without impeding deformation. As such, constraints were only applied to the frontal bone and all faces were fixed, except the internal faces of the proposed craniotomy site. This left the proposed medical device completely free and unconstrained to deform as it would *in situ*. All loads applied in simulation were applied directly to the probe housing. This force was shared equally between the four baffles incorporated into the structure, to mimic the torque provided by the tightening clip. One of the most important aspects of the simulation definition was the generation of friction between the probe housing and craniotomy site. The site itself was designed as the exact female part to the device’s thread. Although a simplification, this has enabled the inhibition of interference of the two components but ensured contact between them. In testing, a coefficient of friction of 0.9 was used as guided by the literature.^43^

### Acquisition of Spectral Data

Acquisition of probe data was made using the InPhotonics Raman probe II, an InPhochelle charge coupled device spectrometer with 785 nm laser and the device detailed. The probe consisted of permanently aligned combination of two single fibers (100 μm excitation fiber, 200 μm collection fiber) with filtering and steering micro-optics, NA 0.22, with stainless-steel cabled fiber with a design to filter laser line and quartz spectral contributions from both input and output fibers (O.D. > 8 at laser line). Samples were loaded into the holder internal to the skull model with the probe aligned *via* the clip mechanism. The spectra were acquired at a laser power of 20 mW and 20 s acquisition time with 15 accumulations at 7.5 mm distance of the probe from the sample. MATLAB R2019a was used to resample and truncate the probe spectra. Negative least squares fitting, against each average spectra using lipid data from ref (44) loaded as components into the analysis tool in WiRE are detailed in refs (39a) and (39b). Processing of spectra was performed in WiRE 5.1, with cosmic rays removed from each scan using the nearest neighbor method, followed by the baseline subtraction using the “intelligent spline” fitting with 11 nodes.

### Data Processing and Multivariate Analysis

Multi-variate analysis was performed using the SKiNET based on SOMs, described in detail in refs (39a) and (39b). SOMs are a single-layer artificial neural network that are represented as a 2D hexagonal array of neurons. Inspired by the visual cortex in the brain, the SOM is trained so that neighboring neurons activate according to similar inputs, in this case, Raman spectra. Each neuron has a weight vector with length equal to the number of variables in a spectrum. Through exposing the network to training samples over a number of iterations, the weights are gradually adjusted to be similar to the input data, so that each neuron only activates on a given spectral signature. The result is a projection of hyperspectral data into 2D space that can be shown as visible clustering according to tissue type and injury state. SKiNET employs the SOMDI, which appends a set of label vectors to each neuron and allows us to study the most prominent features that cause the activation of a particular neuron to a class label. Subsequently, a supervised learning step is introduced to optimize the network, and the class label associated with each neuron is used to quickly identify new data presented to the SOM, allowing for diagnostics. Raw component spectra from brain specific lipids were fitted to SOMDI for a particular state, constituting a physically realistic fit, as our Raman spectra represent a mixed state of positive contributions from constituent components. The change in fitting coefficients were used to interpret the compositional changes to tissue in response to the injury.

## Results

**Figure 1:**
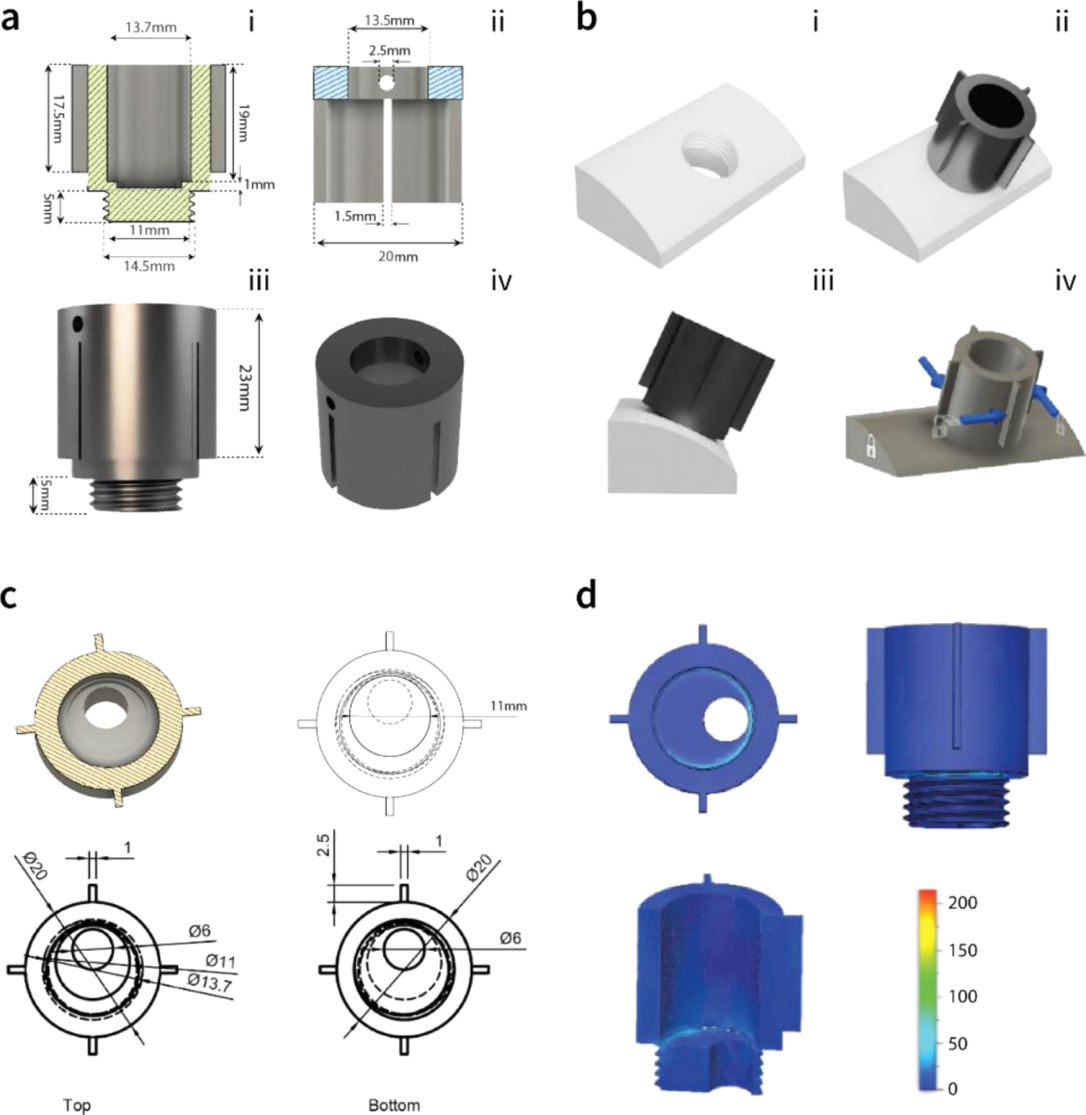
Probe design. (a) Clip is used to aid device positioning and probe alignment: (i) cross-sectional analysis of the Raman probe housing and thread. Note the dimensions of the screw thread and the relation to site parameters detailed in Table 1. (ii) Cross-sectional analysis of the clip and baffle slots. (iii) Tightening and alignment clip fitted to the probe housing. (iv) Aerial view of the clip. (b) *In situ* analysis of the bolt: (i) approximation of the frontal bone at Kocher’s point. (ii,iii) Top and side views of simulation. (iv) Implementation of load and constraints. (c) Section analysis of the housing (left) to demonstrate the internal ledge and drawing of top view of the probe housing (right), with the dimensions of the ledge indicated. (d) Points of high stress in ABS and fracture propagation at the bolt joint. The legend is indicative of the von Mises stress developed in the structure.

The initial device development was orientated around the physical constraints of the probe and cranial access device dimensions (Table 1). The craniotomy site is typically created *via* a disposable clutched perforator drill bit.^45^ In order to prevent the surgeon from drilling into the tissue, the drill should allow for an internal ledge. This also will provide a change in geometry throughout the site. Because of the potentially raised ICP, the design should also consider variations in the degree of brain swelling. A higher ICP means the brain would occupy a greater volume within the calvarium and sub-dural spaces. Therefore, design should include a mechanism to enable fine tuning of the device position to preserve the working distance of the probe and maximize the signal quality. Given these constraints, several key factors need to be taken into consideration upon designing the probe including, the fine-tuning of probe working distance from the brain, secure and safe positioning within the calvarial bone, (Table 1 and Figure 1) alignment of the probe within the device, closure of the brain to the external environment, material selection, which ensures biocompatibility while avoiding fracture, facile, cheap, and repeatable manufacturing and the quantification of design through FEA.

**Table 1:** Probe and Craniotomy Site Design Specifications, Shown in Three Dimensions in Figure 1 for Visualization of How the Constraining Parameters Guided the Device Design^a^

Raman probe parameter	value	site parameter	constraining value
external diameter (mm)	13.7	upper internal diameter (mm)	14.5
probe length (mm)	150	lower internal diameter (mm)	11.0
working distance (mm)	7.5	total length of site (mm)	7.0
laser wavelength (nm)	785	length of safety ledge (mm)	2.0

a The upper and lower site diameter inform the major and minor diameter of bolt, respectively, and constraints on site length in conjunction with variability in brain swelling inform its length [Figure 1a(i)].

A FFF 3D printer was exploited for the prototyping because of its reliability, safety, low cost, and simplicity.^46^ Thermoplastic polylactide was utilized as a biocompatible filament and AF360 software for design (Figure S1). The initial design targeted fine-tuning and preservation of the probe’s working distance. A self-tapping 3D printed thread was used to enable fine adjustment to the position of the probe within the craniotomy site.

This was combined with a supportive housing to prevent damage. However, the probe support yielded an apparent instability. Given that skull is not a planar surface and the added mass of the support increases the moment around the bolt, a simplification of the design was subsequently implemented, and the support was removed. This enabled safe and secure positioning within the frontal bone while minimizing the resource intensity. Further optimization of secure positioning within the frontal bone as well as alignment of the probe within the device was achieved *via* integration of the bolt with an alignment clip. Integration of the components was aided *via* the addition of baffles on the external surface of the holder (Figure 1). Through the incorporation of two M2.5 × 0.35 screws, the clip acted as both a tightening mechanism and a method for alignment (Figures 1c and S1).

To minimize the risk of infection, isolation of the brain to the external environment is crucial. This was tackled by the fashioning of a ledge internal to the holder (Figure 1c) and inclusion of a sunken template for the insertion of a 10 mm diameter quartz disc. Quartz is an ideal candidate material for this purpose because of the little Raman signal at 785 nm (enhanced by its location, far out of focus, and immediately next to the probe) and lack of photoluminescence and transparency.^47^

To fully quantify the performance of the design and the behavior of the bolt *in situ*, FEA provided insightful simulation of the mechanical properties of the frontal bone, the curvature of the skull at Kocher’s point (the traditional default position for intracranial access) and friction between the two bodies (Figure 1b). The simulation also enabled the identification of points susceptible to fracture, optimization of material selection, and design parameters (Figure S2 and Tables S1–S3). The key performance indicators (KPIs), which guided the design optimization and enabled the interpretation of the simulation results are summarized in Table 2, where the maximum displacement difference (MDD) quantifies the degree of fracture and minimum safety factor (SF) quantifies the risk of fracture and device failure.^48^

**Table 2:** KPIs Used in Simulations

KPI	equation	simulation type
MDD	max (housing displacement) – min (bolt displacement)	event simulation
SF	yield stress/working stress	static stress

We have used various event simulations to investigate the effects of material selection on the basis of a thread length of 7 mm (Figure S2 and Tables S1–S3). The key indicators of design optimization included the minimization of heterogeneous deformation or displacement across the device and the mitigation of the device fracture. The materials investigated in simulations included ABS, polyamide 6 (PA6), and titanium 6AI-4V. ABS was chosen for its properties as a thermoplastic, which are similar to the prototyping material, that is, the PLA and biocompatibility.^49^ Nylon 6 can be further considered as a potential material given the recommended use in medical devices with short-to-medium term contact applications in conjunction with its material properties.^50^ Titanium is a renowned biomedical material, primarily for its effective integration with tissue, reducing fibrous tissue growth and scarring around implants.^51^ Combined with the modern stereo-lithography methods, device fabrication should take advantage of both the biocompatibility and strength of the material for a given application.^52^ Outputs from the event simulations are summarized in Table 3, where the maximum variance of displacement is defined as the difference between the displacement of the thread and probe housing and quantifies the degree of crack propagation at the joint. From the simulations, it was established that titanium poses little risk of fracture even under loads of 1.6 Nm. On the other hand, both ABS and PA6 exhibit fracture at lower loads, with PA6 deforming less than ABS.

**Table 3:** Event Simulation Outputs and Minimum SF Associated with Static Stress Analysis^a^

material	max. variance displacement (μm)	max. stress at fracture (MPa)	torque at fracture (Nm)
ABS	2828	45.16	0.8
PA6	832	70.74	0.96
titanium	86	N/A	N/A

simulation	thread length (mm)	min. SF
SS-7	7	2.60
SS-8	5	3.32

a Comparative analysis in thread length is made with respect to simulations with ABS as the device material.

**Figure 2:**
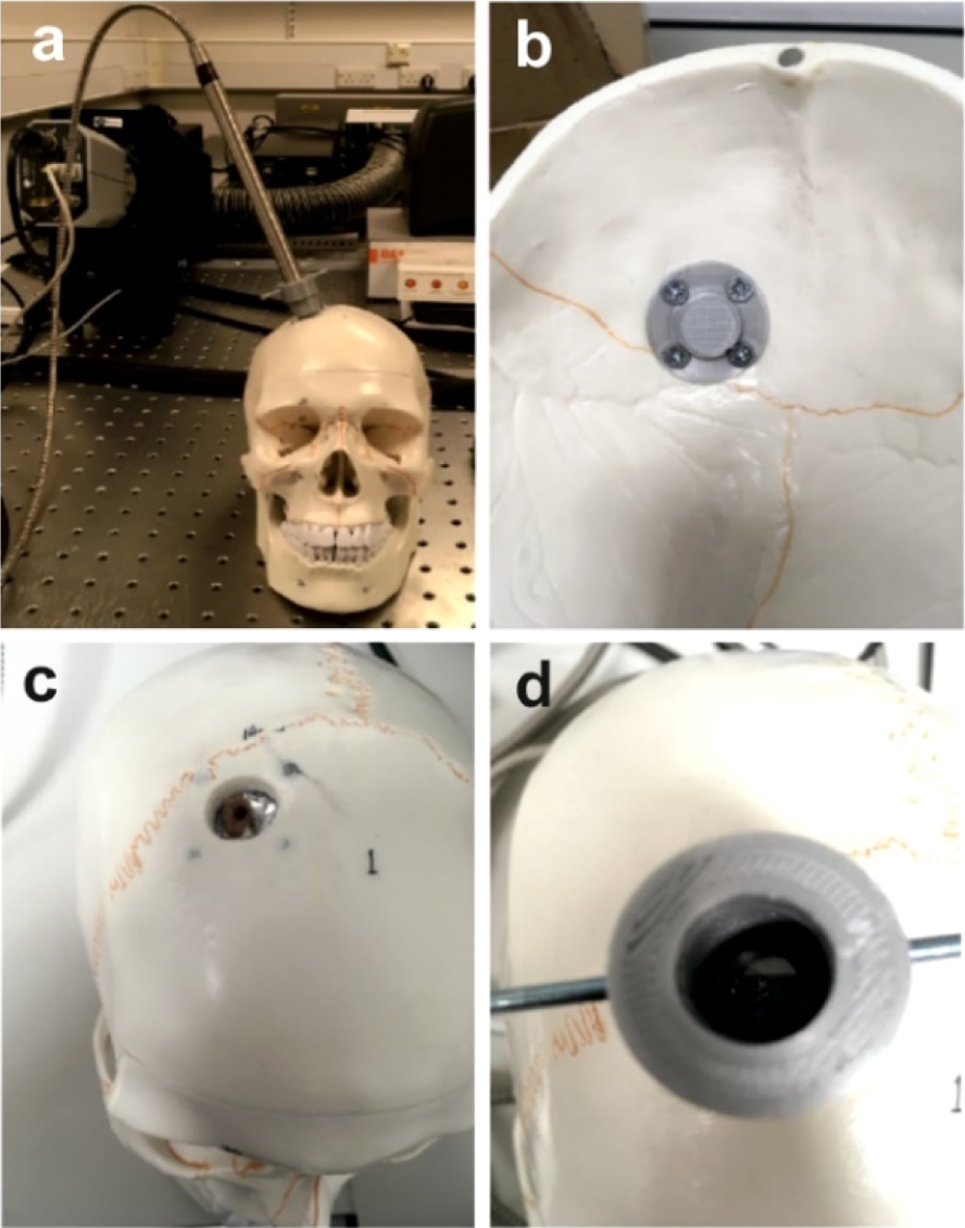
Development of a skull model. (a) Fully assembled model. (b) Sample holder internal to the skull. (c) Sample holder at Kocher’s point. (d) Medical device *in situ*.

In the early prototyping and development of the skull model, it became apparent that the internal ledge formed in the craniotomy procedure would provide an obstruction to implementation of the device even once removed. Fracture of device prototypes was often observed with complex skull curvature, and friction between the thread and skull is hypothesized to be the major factor. Hence, we used the static stress analyses to investigate the effect of thread length, which is equivalent to varying the area for frictional contact *in situ*. Thread lengths of 5 and 7 mm were investigated, with the step change between the two, equivalent to the thickness of the internal ledge, as detailed in Table 1. In all simulations, ABS was chosen as the device material (Figure 2 and Table 3).

The equivalent of 0.8 Nm and a minimum of SF > 1 indicates the device is safe for general application. However, a minimum of SF ≥ 4 is typically recommended in the literature to minimize risk in medical applications.^53,54^ Upon static stress analysis of the 5 mm thread length using titanium 6AI-4V, a minimum SF of 11.58 was achieved. The points of greatest induced stress and fracture propagation are shown in Figure 1d. These results indicate that the ABS is not an optimal choice for future design; however, PA6 and titanium are found to be both suitable materials for this purpose. For the in-patient utilization of these materials, further optimization should consider material selection in the scope of treatment viability, device utility, and cost.

Subsequent to the prototype design, the setup was tested for its potential to act as a clinical *in vivo* device. A skull model was developed through the creation of a craniotomy site *via* conventional neurosurgical tools, 3D printing, and insertion of a sample holder internal to the calvarial vault (Figure 2). The device with the probe was then inserted into the site of interest, and the *in situ* measurements were carried out.

**Figure 3:**
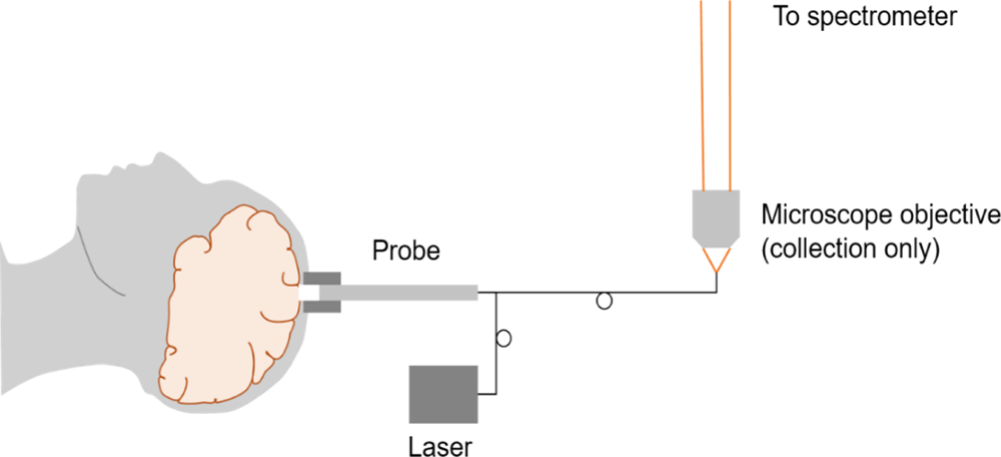
Performance of the device. Validated by collecting the output signal from the distal end of fiber using the spectrometer as a detector and the probe arrangement using the skull model.

Raman spectra were acquired using the experimental geometry, as shown in Figure 3, which allowed coupling the output fiber of the probe to a portable Raman spectrometer. In this configuration, the spectrum was collected by focusing onto the output of the probe collection fiber (held axially using a 3D printed holder) and a Raman acquisition sequence was run using the WiRE software. The diameter of the probe (Table 1) being smaller than that of the bolt enables it to be easily incorporated into a standard of care (Figure S3).

**Figure 4:**
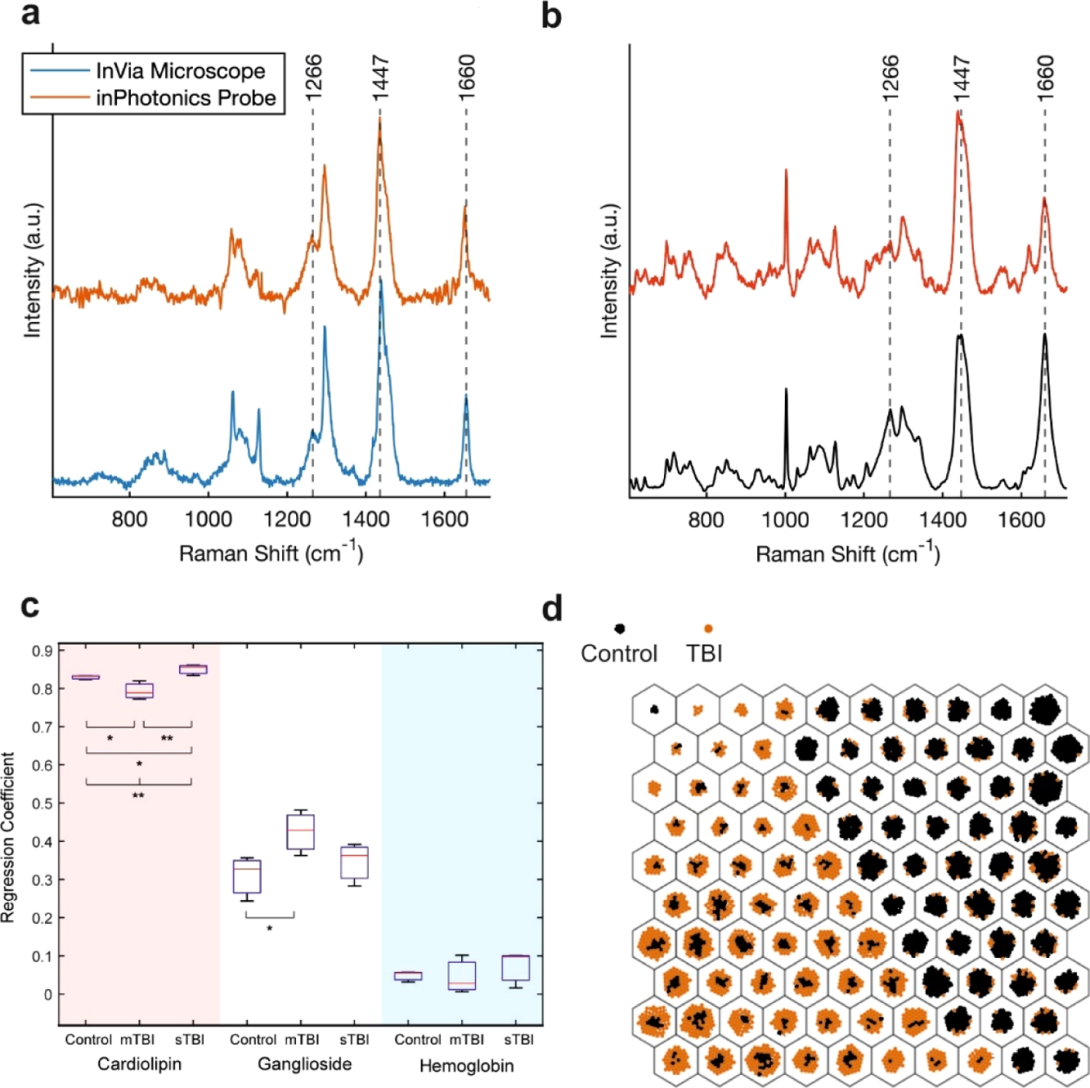
Spectroscopic performance. (a) Raman spectra from the developed intracranial spectroscopic setup with coupled output fiber from the probe (red) *vs* the microscope objective (blue), allowing measuring the performance of the probe and the skull model. (b) Representative spectra from TBI (red) compared to the healthy controls (black) in murine brain tissue (785 nm, 20 mW, 3–5 s) with specific bands highlighted at 1266, 1447, and 1660 cm^–1^ and (c) changes to relative lipid composition as a result of severe (sTBI), moderate (mTBI) *vs* control.^39^ The boxplots show the non-negative least squares regression coefficient fitted to the average spectrum collected from each sample (*p* < 0.05). (d). SOM clustering of Raman spectra from for TBI (red) and control (black) cohort.

Using this setup, we successfully demonstrate that spectra obtained through the developed device design reliably reproduce the information measured from commercial Raman microscopes (*e.g.*, inVia Renishaw) (Figure 4a). The effects of fluorescence in the early acquisition of spectra, under typical exposure and laser power (5–25 s, 20–30 mW), were minimized through the development of a specific acquisition sequence in combination with the optimization of the probe’s distance from the sample (Table 4). Data collection was carried out *via* the Raman setup in conjunction with the bolt and skull model, and the key considerations which guided the correct parameter selection were determined by the maximum achievable signal-to-noise ratio and a minimum risk of laser damage to sample, which enabled collection of a large number of high quality of spectra with a negligible effect of fluorescence through the utilization of a longer wavelength source.

**Table 4:** Acquisition Parameters of the Probe

parameter	probe value
spectral domain (cm^–1^)	–200 to 3723
laser wavelength (nm)	785
laser power (mW)	20–30
exposure (s)	5–25
accumulations	15
probe distance from sample (mm)	7.5 (equal to working distance of the probe)

Building upon our recently established murine model of direct cortical TBI impact,^39a,39b^ demonstrating the differentiation between TBI, the healthy controls amongst differing states of injury severity, the measured key bands of murine tissue used to simulate the spectral response from the brain, show corresponding changes with TBI including dominant bands at 1266, 1447, and 1660 cm^–1^ (Figure 4a,b), which are attributed to cellular metabolic distress and dysfunction. These bands are representative of C–C aromatic ring *stretchin*g associated with fatty acids that comprise certain lipids in the brain, the *in-plane bending* of CH_2_ and CH_3_ (*asymmetric*) bonds present in lipids and proteins constituting what is likely to be gray matter and the C=O and C=C coupled bond *stretching* in unsaturated fatty acid residues, respectively.^44,55^ Changes to lipid composition in response to TBI (post-fixed tissue, 3 days after injury) determined by non-negative least squares fitting have shown previously that the coefficients for each lipid spectrum were proportional to the concentration measured within each sample,^39b,44^ with the main statistically significant differences arising from cholesterol and cardiolipin, where a reduction in the latter is linked to the decrease in bands at 1266 and 1660 cm^–1^ for TBI. The regression coefficients for the cardiolipin, ganglioside, and hemoglobin (Figures 4c and S4) show insights into the separation of injury classes, with a larger coefficient indicating a greater contribution to the spectra. It is of note that the coefficients related to mild TBI observe a greater variance given the complexities and natural variation of biochemical response associated. Further to the discernible differences in peaks from Raman spectra, machine learning algorithms were applied to extract the subtle spectral changes present. Short acquisition times, representing the real-world conditions, meant that individual spectra used as training inputs exhibited only *minute* changes, which *via* SKiNET were used to accurately identify TBI versus control groups (Figure 4d), where the neurons are grouped according to particular features, with each hexagon in the SOM being colored according to the type of data it activates, that is, control (black) versus the TBI (red), providing visualization on the data classification. The preformed fitting of raw component spectra from brain specific lipids correlates to the SOMDI for a particular state (Figure S4a,b). The subtle changes identified *via* SKiNET to spectral features in the analysis of Raman spectra show a clear separation between TBI and control groups (Figure 4d).

## Discussion

In TBI disease state, the relative ratio of the 1447/1660 cm^–1^ peak height is found to be considerably increased and correlating with the injury severity (Figures 4b, S4, and S5). Previous studies have demonstrated that white matter presents a broad Raman shift in the region of 1200–1350 cm^–1^ with similar normalized intensity to the 1660 cm^–1^ peak, whereas gray matter does not.^56^ However, the peaks were often much lower in intensity relative to the 1447 cm^–1^ band. It is therefore reasonable to note that an important confoundment to the findings relating to 1447/1660 cm^–1^ ratios could be the model itself. The direct cortical injury model is an energy-focused model, with increasing energy of strike to the cortical surface. A possible explanation to not seeing a progressive difference in the height of these peaks is that the surface injury from each strike (regardless of energy) inflicts similar injury to the brain surface (injury saturation in effect). A focal surface measurement in these cases may not be ideal.

However, a one-way analysis of variance analysis shows a statistically significant difference in the contribution from cardiolipin compared to the control (Figure 4c), which is linked to the increase in the ratio of bands at 1447 and 1660 cm^–1^ in Figure 4b in TBI for both moderate and severe TBI versus the control group. A statistically significant change is also observed between injury severity for cardiolipin and exists in ganglioside for mTBI compared to control. However, there is no statistically significant difference between mTBI and sTBI in this case. Small coefficients were fitted for hemoglobin for sTBI and mTBI but were not statistically significant. The hemoglobin change is most probably a result of hemorrhage, yielding an increase in its concentration specific to the injury site. It has been reported that cardiolipin undergoes oxidation during the pathophysiological cascade in TBI, with an accumulation of similar oxidation products in the region of injury.^57,59^ An accumulation of ganglioside in the region of injury has also been demonstrated.^42^ Temporal changes from direct analyses of brain tissue have been previously studied by Surmacki *et al.*(58) using the murine TBI model. Cardiolipin, playing a key-role in cell metabolism and signaling, has been shown to compromise the blood brain barrier, triggering the metabolic disruption and the biochemical cascade following TBI cell damage, resulting in its release.^60^ These results are further consistent with study by Tay *et al.*, where the authors identified a link between the observed spectral changes and apoptosis *via* comparison to immunohistochemistry of TBI in mice using Raman spectroscopy.^33^ Consistent changes are observed to spectra in response to injury, and particularly, in the 1266 cm^–1^ relative to the 1447 and 1660 cm^–1^ bands, which are proportional to injury severity (Figures S4 and S5). These findings are in correspondence with the subtle changes seen in the previous study by Surmacki *et al.* Similar peaks were identified as strong SOMDI weights as derived from analysis provided by SKiNET (Figure 4d). These weights are associated with each group at 1003, 1266, 1337, 1447, and 1660 cm^–1^ representing, skeletal C–H of phenylalanine, C–C bending of mixed proteins/lipids, C–N stretching, N–H bending of the amide III, C–H_2_ bending, and C=C stretching of the proteins and lipids, in correspondence with the Figures S4–S6 and the literature.^33,39,42,56−59^

It is worth noting that invasive intracranial monitoring devices tend to be used within the context of severe TBI where there is a clear indication for neuro-protective sedation. Typically, these are used in the acute phase of injury in which invasive monitoring takes place and is considered to be between 3 and 5 days. However, there are common exemptions, and a number of devices are licensed to be *in situ* for up to 2 weeks. Our device would be analogous to incorporate the current standard of invasive monitoring care. This would be inserted at presentation and remain *in situ* during the acute phase of injury evolution (between 2 and 7 days depending on secondary injury evolution). The accumulation of inflammatory debris might play a factor at the optical interface. To mitigate this, a saline flush, representing a reflective isotonic environment, a self-cleaning conduit, or mechanical (iris pattern open and close) sweep can be integrated into the design such as those used in cerebral and ventricular endoscopes, combined with poly(tetrafluoroethylene) and or other anti-adhesion coatings. A further possibility will be to use an anti-fouling coating, such as for instance the Surphys 009, which has been previously shown to considerably reduce a stent bacterial counts compared to controls with decreased pathogen adherence.^60^ Light absorption by water in the wavelengths described in our study is minimal; therefore, tissue heating at this irradiance using the 785 nm laser, the laser power, and the exposure time (Table 4) will cause negligible if any tissue damage. Similar intensities of light are already used in direct to tissue oxygen tension, including for instance, the Raumedic PbtO_2_ measurements *via* fluorescence quenching, with a safety profile similar to other invasive monitoring modalities.^61^

## Conclusions

We have designed, engineered, and characterized a novel proof-of-concept device to *in situ* track the biochemical changes of brain composition under the pathophysiological cascade associated with TBI *via* Raman spectroscopy. Titanium 6AI-4V has been established as a preferable material of fabrication with the final design consisting of an M15 × 1.5 thread of 5 mm in length adjoined to a probe housing. Static stress analysis of the design, when printed in titanium 6AI-4V, revealed a minimum SF of 11.58. The utilization of this material has obvious performance and biocompatibility benefits; however, further optimization should be considered for material selection in the scope of treatment viability, device utility, and cost. A skull model has also been developed, which has highlighted the potential for clinical translation of the design in view of complex skull curvature. Spectra obtained from the design setup accurately reproduce those acquired with the standard commercial Raman system, and the collected signal demonstrates that they are comparable in terms of quality and the detected bands to the TBI model, reliably retrieving subtle, specific biochemical changes to those typically measured using the commercial Raman microscope.

This proof-of-concept device provides a clear demonstration that Raman spectroscopy of brain cortex has the potential to provide an added value within the context of clinical monitoring and improved management of TBI. A great deal of investigation was carried out to quantify properly the use of the Raman spectra to stratify injury severity and resolve the evolution of pathology prior to any form of clinical testing or introduction; however, we demonstrate here the concept in its most basic form. It also lays a platform for further developments of probe design *via* for instance, including an ICP monitor with other standards of care, to aid clinical integration and translation of the device into clinic by removing the need for any further intracranial access burden. Ultimate integration of such a tool with emerging artificial intelligence techniques, such as our recently developed SKiNET method will further provide important interpretable therapeutic and management guidance. Real-time, rapid *in vivo* spectroscopic brain measurements in TBI patients will enable establishing insights into biological pathways underlying the associated neurological pathophysiology and could conceivably allow tracking the passage and dosage of pharmacological therapeutics and anesthetics.

## Supporting Information Available

Device design and mechanical drawings, detailed simulation parameters and system losses as well as SOMDI classification and regression fitting and colored Raman maps of the ratios/peak intensities (PDF)
