# Supplementary material for: Development and Characterization of a Probe Device toward Intracranial Spectroscopy of Traumatic Brain Injury
Source: ACS Biomater Sci Eng. 2021 Feb 22;7(3):1252–62. doi: 10.1021/acsbiomaterials.0c01156 (PMC7944476; doi:10.1021/acsbiomaterials.0c01156)
Supplement: Supplementary file 1 — ab0c01156_si_001.pdf [file ab0c01156_si_001.pdf]

-SUPPORTING INFORMATION-

Development and Characterization of a Probe-Device Towards Intracranial Spectroscopy of Traumatic Brain Injury

*Max Mowbray[1], Carl Banbury[2], Jonathan J. S. Rickard[2, 4], David J. Davies[3] and Pola Goldberg Oppenheimer[2, 5, *]*

* Corresponding E-mail: GoldberP@bham.ac.uk

5 Pages

6 Figures

3 Tables

Figure S1………...S2

Figure S2………...S2

Table S1………...S3

Table S2………...S3

Table S3………...S3

Figure S3………...S3

Figure S4………...S4

Figure S5………...S4

Figure S6………...S5

S1. Final Device Design and Mechanical Drawings

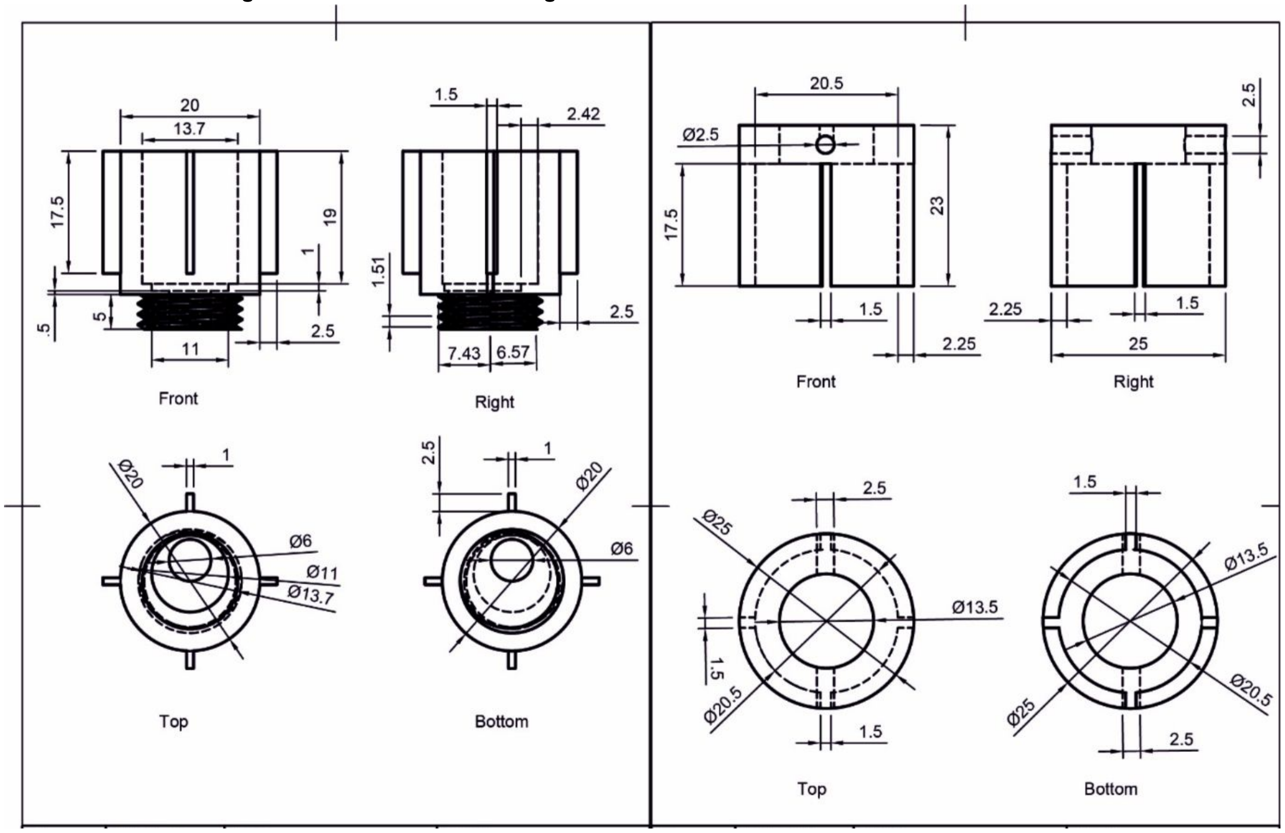

Figure S1-Sn. Probe design Iterations and details.

S2. Simulation Parameters

S2.1 Constraints

All comparative simulations proceeded on the basis of the same constraints and load. In AF360, implementation of constraints is done to ensure the static stability of the model, without impeding deformation (Autodesk Fusion 360). As such, constraints were only applied to the fontal bone. All faces were fixed, except the internal faces of the craniotomy site. This allowed the medical device completely free and unconstrained to deform as it would *in-situ*.

S2.2. Loads

All loads applied in simulation were applied directly to the probe housing. This force was shared equally between the four baffles incorporated into the structure, to mimic the torque provided by the tightening clip (Fig. S2).

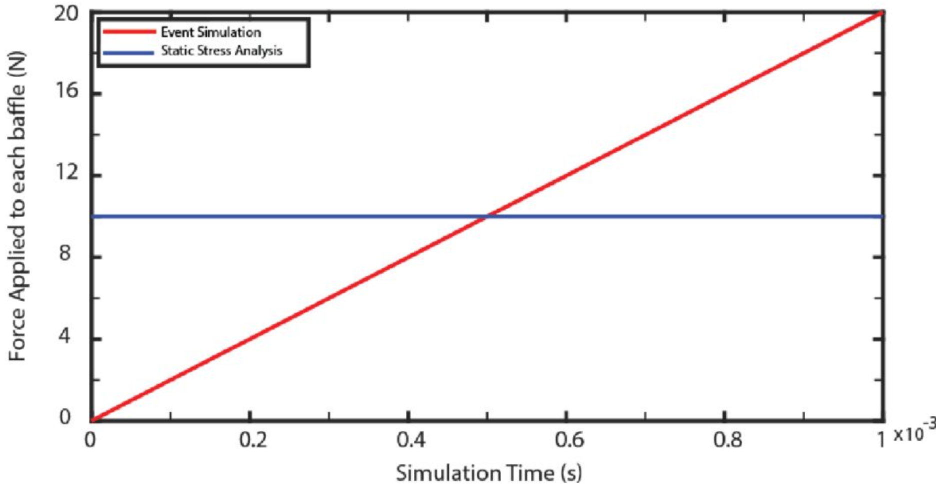

Figure S2-Sn. The loads used in event simulation and static stress analysis.

S2.3 Contacts

One of the most important aspect of simulation definition was the generation of friction between the probe housing and craniotomy site. The site itself was designed as the exact female part to the device's thread. This enabled inhibition of interference of the two components but ensured contact between them **(Table S1-Sn)**.

Table S1-Sn. Implementation of contacts during simulation. A coefficient of friction (CoF) of 0.9 was adopted from ref. 59..

Study	Contact Type
Event Simulation	CoF = 0.9 with self-contact
Static Stress (S# -#)	Bonded

S2.4 Mesh Generation

The same mesh parameters were implemented for all studies to ensure an accurate comparison. The key parameters are detailed in **Table S2-Sn** and were selected to maximise accuracy, whilst reducing computational intensity.

Table S2-Sn. Key meshing parameters.

Parameter	Value
**Average Element Size (% of part size)**	7
**Element order**	Parabolic
**Minimum Element Size (% of average size)**	20
**Curved Mesh Elements**	Enabled

S3.1 Investigation of System Losses

Table S3-Sn. Quantification of power losses in the system.

Laser Power Parameter	Power (mW)
**Software Setting**	500
**Power at source**	100
**Power at point of probe emission**	20

S3.2 InPhotonics Raman Probe II: Focusing and Filtering Optics

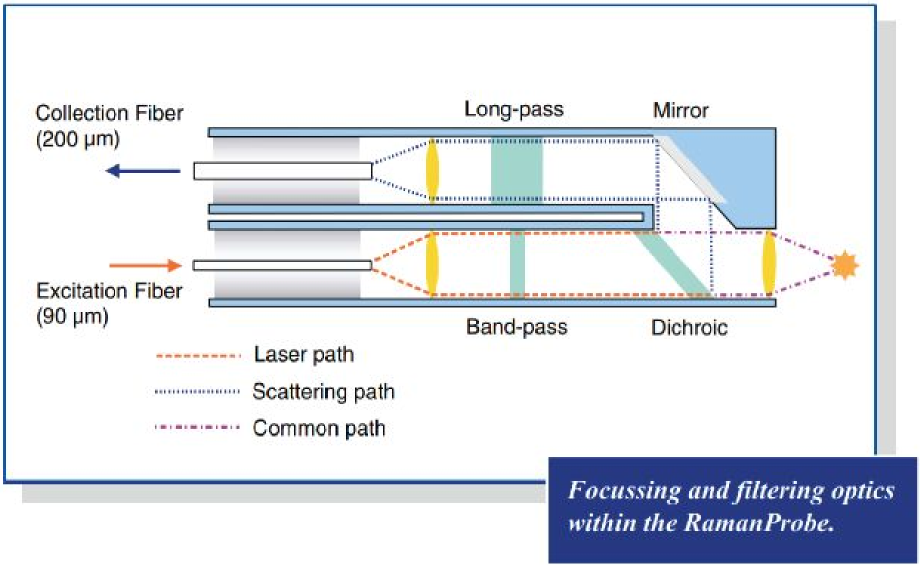

Figure S3-Sn. Inherent coaxial design within the probe minimises background due to the probe fibres.

S4. SKiNET and SOMDI Classification and Regression Fitting of TBIs *versus* Controls

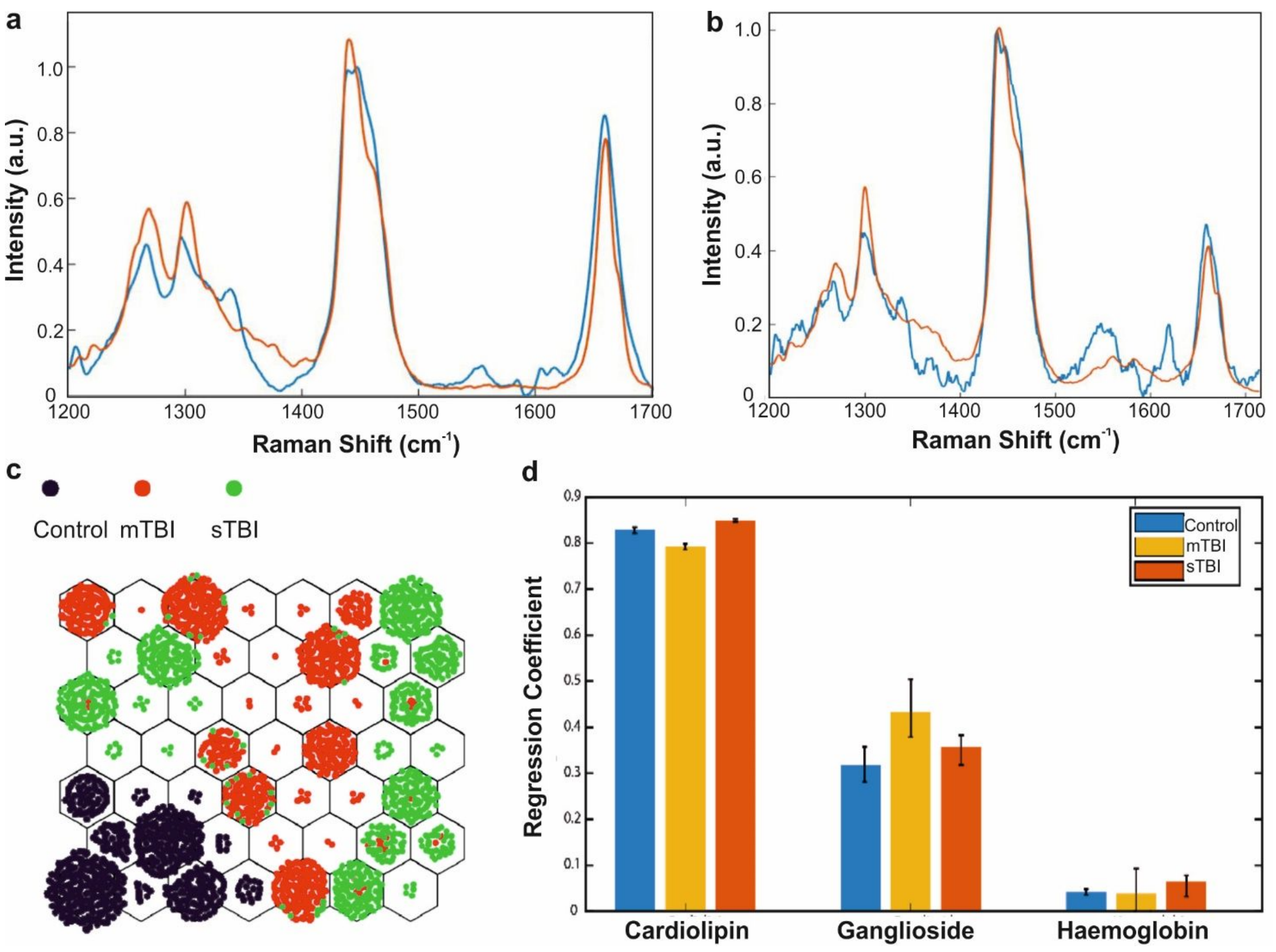

Figure S4-Sn. Non-negative least squares fitting *versus* SOMDI for **(a)** control and **(b)** TBI tissue. **(a-b)** Features extracted (SOMDI) from SOM shown in **Fig. 4d**, highlighting the Raman bands most influential to neurons in the SOM for control and TBI groups *versus* regression fitting results with a high SOMDI score associated with wavenumbers that strongly influence clustering **(c)**. Fitting was performed in the range 1200-1714cm^{-1} to identify the relative contributions in each tissue sample for uninjured, mTBI and sTBI. The resultant fitting coefficients for each lipid spectrum are proportional to the lipid concentration measured within each tissue sample. **(d)**. Changes to lipid composition as a result of TBI. [39] The preformed fitting of raw component spectra from brain specific lipids correlates to the SOMDI for a particular state.

S5. Representative SOMDI and Coloured Raman Maps of the Peaks Ratio at 1447/1660cm^{-1}

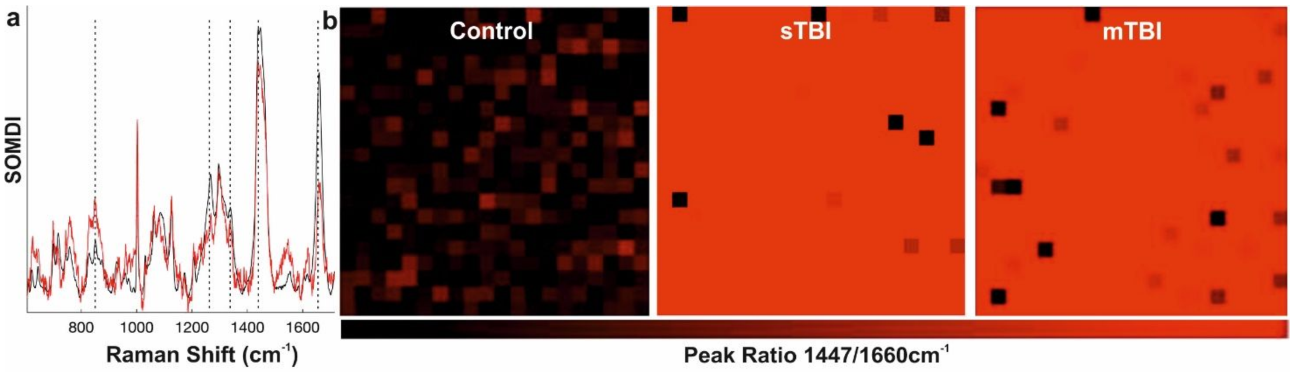

Figure S5-Sn. (a). Features extracted (SOMDI) from SOM highlighting the Raman bands most influential to neurons in the SOM for control and TBI groups. **(b).** Coloured Raman maps of the average ratio of the peaks at 1447 *versus* 1660cm^{-1} for uninjured (left), sTBI (middle) and mTBI (right) groups. Consistent changes observed to spectra in response to injury in the 1447cm^{-1} *versus* 1660cm^{-1} bands proportional to injury severity are in correspondence with the subtle changes seen in the previous study by Surmacki et al. [34,38]

S6. Coloured Raman Maps of the Ratios/peak for Individual Groups of Uninjured, sTBI and mTBI

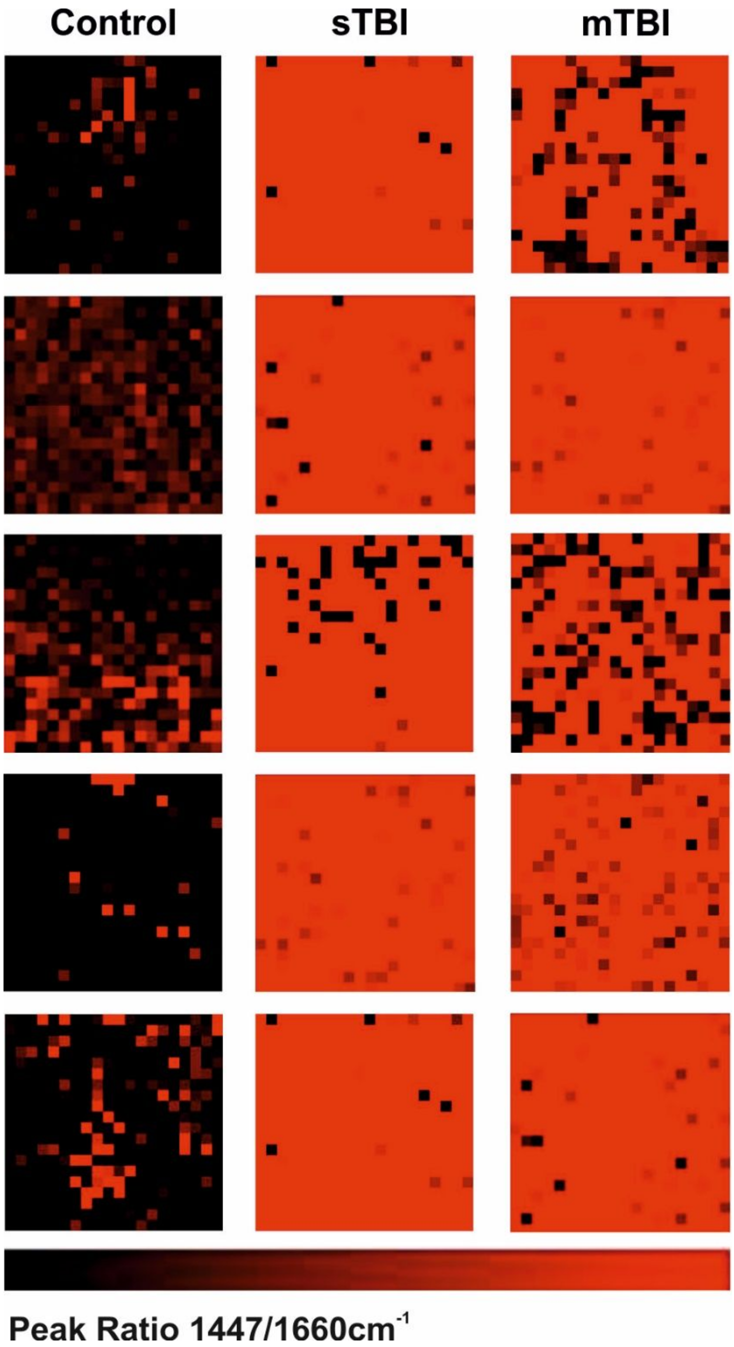

Figure S6-Sn. The corresponding Raman maps, representative of five individual animals for uninjured controls (left), sTBI (middle) and mTBI (right).The band at 1447cm^{-1} is associated with the C-H_2 stretching and 1660cm^{-1}, assigned to C=C stretching, is commonly associated with amide groups in lipids and proteins.
